# Factors Affecting the Adsorption of Heavy Metals by Microplastics and Their Toxic Effects on Fish

**DOI:** 10.3390/toxics11060490

**Published:** 2023-05-28

**Authors:** Qianqian Chen, Haiyang Zhao, Yinai Liu, Libo Jin, Renyi Peng

**Affiliations:** Institute of Life Sciences and Biomedicine Collaborative Innovation Center of Zhejiang Province, College of Life and Environmental Science, Wenzhou University, Wenzhou 325035, China; 21451335004@stu.wzu.edu.cn (Q.C.); 20160106@wzu.edu.cn (H.Z.); 21461338012@stu.wzu.edu.cn (Y.L.)

**Keywords:** microplastics, heavy metals, fish, toxic effects, regulatory mechanisms

## Abstract

Fish not only constitute an important trophic level in aquatic ecosystems but also serve as an important source of protein for human beings. The health of fish is related to the sustained and healthy development of their entire aquatic ecosystem. Due to the widespread use, mass production, high disposal frequency, and degradation resistance of plastics, these pollutants are released into aquatic environments on a large scale. They have become one of the fastest growing pollutants and have a substantial toxic effect on fish. Microplastics have intrinsic toxicity and can absorb heavy metals discharged into water. The adsorption of heavy metals onto microplastics in aquatic environments is affected by many factors and serves as a convenient way for heavy metals to migrate from the environment to organisms. Fish are exposed to both microplastics and heavy metals. In this paper, the toxic effects of heavy metal adsorption by microplastics on fish are reviewed, and the focus is on the toxic effects at the individual (survival, feeding activity and swimming, energy reserves and respiration, intestinal microorganisms, development and growth, and reproduction), cellular (cytotoxicity, oxidative damage, inflammatory response, neurotoxicity, and metabolism) and molecular (gene expression) levels. This facilitates an assessment of the pollutants’ impact on ecotoxicity and contributes to the regulation of these pollutants in the environment.

## 1. Introduction

**Figure 1:**
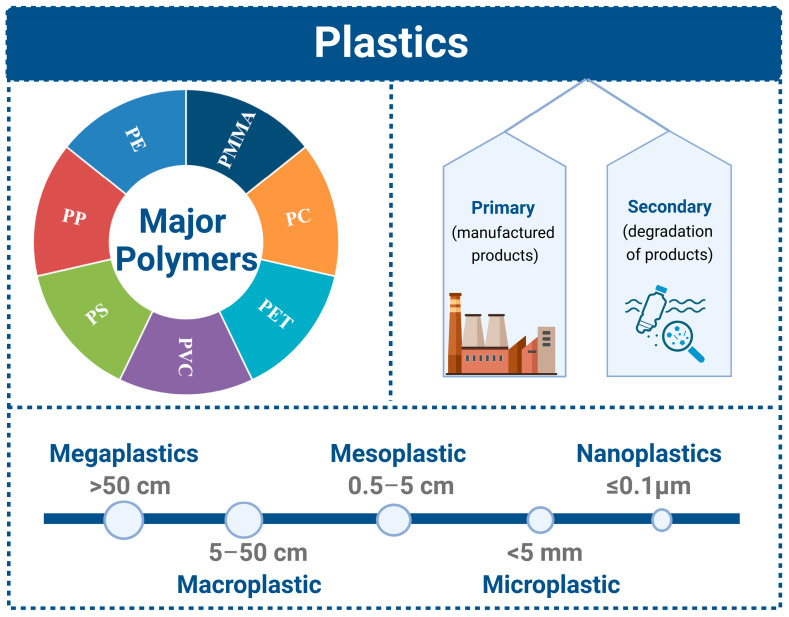
Definitions of plastics. PS (polystyrene), PE (polyethylene), PP (polypropylene), PVC (polyvinyl chloride), PET (polyethylene terephthalate), PC (polycarbonate), and PMMA (polymethyl methacrylate).

A large number of plastic products are discarded after use, resulting in the accumulation of large amounts of plastic waste in the environment. Under the combined influence of physical, chemical, and biological environmental factors, the degradation of plastics produces different forms and sizes of debris: nanoplastics (≤0.1 μm), microplastics (<5 mm), mesoplastics (0.5–5 cm), macroplastics (5–50 cm), and megaplastics (>50 cm) [1,2,3]. There are many kinds of microplastics (MPs), and they are often divided into primary MPs and secondary MPs according to their production methods [4]. Primary MPs consist of direct industrial products and artificial plastic particles that meet specific needs. These particles are widely used in daily chemical products as fillers, film-forming agents, friction-inducing agents, and suspension agents, as well as in the processing and molding of textiles and plastic products [4]. Secondary MPs mainly come from the decomposition of industrial plastic products [4]. Plastic products are widely used in various industries and daily life because of their low price, broad applicability, and strong durability (Figure 1) [5,6,7]. Therefore, the application scale and production growth rate of plastics are higher than those of most other man-made materials. Microplastic pollution not only causes enormous damage to fish but also has an incalculable impact on ecosystems.

Compared with MPs, heavy metal pollutants in water do more damage to fish. At present, heavy metals (HMs) are listed as “the number one environmental pollutant” in countries all over the world, and they have a wide range of pollution sources [8,9,10]. Usually, wastewaters from mining, smelting, electroplating, paint production, printing and dyeing, and other industries along with solid waste exudates and domestic sewage enter water bodies [11]. Heavy metal pollution refers to the pollution that results when compounds containing heavy metal ions enter water bodies. According to current research, the metal pollutants mainly include Cd, Co, Cu, Ni, Pb, Zn, and Hg [12,13]. Heavy metal pollutants have low degradability and high toxicity and are widespread. After HMs enter aquatic ecosystems, on the one hand, they can enter the circulatory system of fish through the water to damage fish health; on the other hand, MPs can serve as carriers that transport HMs into fish bodies where they accumulate in the digestive system of fish [14,15,16]. This can cause various kinds of damage to fish and slow their activity, arrest development, hinder reproduction, or even cause mass death, and the HMs can be passed through the food chain and become biomagnified. They gradually accumulate in organisms with higher trophic levels and even endanger human health.

In recent years, combined pollution from MPs and HMs has posed a serious threat to ecosystems, and many scholars have begun to focus on the effects of MPs on the biotoxicity of HMs in the environment [17]. While MPs migrate through water bodies, the heavy metal pollutants that are enriched on their surfaces are transferred to sediments and groundwater along with the MPs, and these pollutants persist in all kinds of water bodies, which may have a lasting impact on natural environments.

Both MPs and HMs are difficult to degrade. When large-sized MPs enriched with HMs are ingested by fish, they are not easily excreted, which prolongs the residence time of these harmful substances in fish intestines [11,15]. However, some MPs with smaller particle sizes dissociate from the adsorbed HMs and enter the circulatory system of fish and may result in dual toxic effects from MPs and HMs. First, MPs cannot be digested and absorbed in fish, and they often lead to lumen obstruction, scratches, and a series of problems, such as asphyxia, digestive tract obstruction, and mechanical organ damage [18,19,20]. On the other hand, chronic injury accompanied by biological life history changes can lead to alterations in appetite, decreases in feeding ability, decreases in risk aversion, and decreases in adaptability to the environment, which can lead to death [21]. Second, MPs are harmful chemicals, and the chemical additives added to synthetic plastics are released when they are degraded by biological or abiotic action, which causes subsequent damage to the physical functions of fish [22]. Third, after MP-adsorbed HMs enter fish, some HMs are released into the organs and tissues of fish, resulting in acute heavy metal poisoning in the fish [23]. Therefore, in the digestive system of fish, the coexistence of MPs and HMs often increases the toxicity of the pollutants to fish.

In view of the fact that both MPs and HMs strongly affect the physiological functions of fish, the toxic effects of MPs with adsorbed HMs on fish still need to be systematically and thoroughly evaluated. This review summarizes the toxic physiological effects of HMs adsorption by MPs on fish. We focus on effects at the individual, cellular, and molecular level to explain the toxic effects of plastic-adsorbed HMs on fish in hopes to provide valuable reference information for the assessment of the environmental pollution effects of MPs combined with HMs.

## 2. Factors Influencing Heavy Metal Enrichment on MPs and Removal Strategies

**Figure 2:**
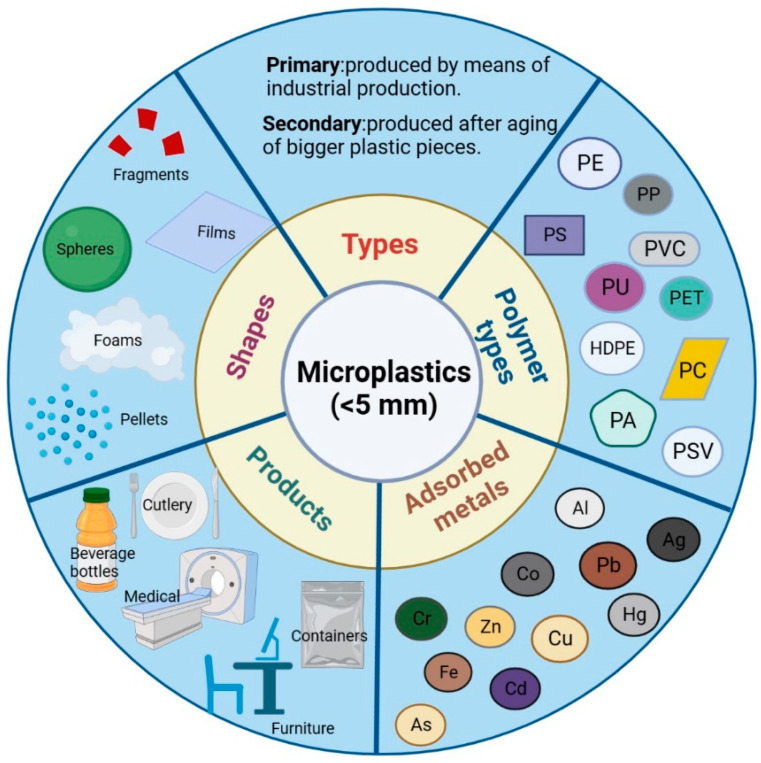
Types, shapes, major polymerytpes, products, and absorbable metals of microplastics. PS (polystyrene), PE (polyethylene), PP (polypropylene), PVC (polyvinyl chloride), PET (polyethylene terephthalate), LDPE (low-density polyethylene), HDPE (high-density polyethylene), PU (polyurethane), PC (polycarbonate), PLA-PA (polyamides), PV (polyvinyl), PSV (plastic second-life polymer), SAN (styrene acrylonitrile resin), PDM (polydimethylsiloxane), Pb (lead), Cd (cadmium), Ni (nickel), Cu (copper), Co (cobalt), Zn (zinc), Cr (chromium), Al (aluminum), Mn (manganese), Fe (iron), As (arsenic), Sb (antimony), Sn (stannum), Ag (silver), Hg (mercury).

The adsorption of HMs by MPs in water involves a variety of mechanisms, which can be divided into two categories: physical adsorption and chemical adsorption [24]. Physical adsorption mainly includes electrostatic interactions and internal and external diffusion, while chemical adsorption mainly involves interaction through complexation and cation-π bonds [24,25]. On the other hand, the HM adsorption mechanism of MPs can include both physical adsorption and chemical adsorption, and it is specific due to the different physical and chemical properties of MPs [26]. In addition, according to the type, size, and surface properties of MPs, their adsorption capacity for the same heavy metal can also be different (Figure 2). The adsorption capacity of MPs for HMs is mainly affected by their own properties (species, surface area, aging status, and functional groups), heavy metal ion properties (species and concentration), and environmental factors (pH and ionic strength). There are differences in the surface charge, functional group, and acid–base characteristics of different types of MPs, which affect their adsorption of HMs.

### 2.1. Influence of the Properties of MPs

Firstly, the properties and structures of the polymers that make up plastics and the particle size and morphology of MPs are important factors affecting the adsorption capacity of MPs. Brennecke et al. found that polystyrene (PS) and polyvinyl chloride can adsorb Cu and Zn, but there are significant differences in the adsorption capacity of different MP materials for different heavy metal ions [27]. Liu et al. found that the properties of MPs affect the interaction between MPs and phthalate esters (PAE). The chemical properties of MPs have an important influence on adsorption. The adsorption order of the three kinds of MPs for PAE is PS > PE > PVC [28]. In addition, PS has a benzene ring substituent in its structure and has a strong π–π interaction with PAE, so it has a large adsorption capacity [28]. Gao et al. found that the adsorption of Pb, Cu, and Cd by MPs (PP, PE, PAL-PA, and PVC) and polyoxymethylene (POM) in marine environments is affected by plastic type [29]. In addition, the metal adsorption capacity of polyvinyl chloride (PVC) and polypropylene (PP) is higher than that of polyamides (PLA-PA), polyethylene (PE), and POM [29]. Moreover, hydrophobicity and diffusivity also affect the adsorption of metal ions by MPs, among which PVC has the strongest adsorption capacity for metal ions because of its polar group (C-Cl), and PP adsorbs metal ions through surface adsorption [29,30]. Therefore, different types of MPs have different adsorption capacities for metal ions, which results mainly from their different surface physicochemical properties.

### 2.2. Effects of Environmental Factors and Properties of HMs

Environmental factors such as light, heat, ultraviolet radiation, mechanical processes, biological processes, radiation, and oxidation reactions can change many properties of MPs, including their pore size distribution, specific surface area, and functional groups [31,32]. Changes in environmental factors can also change the ability of MPs to adsorb HMs. At present, studies have proven that certain changes in the surface of MPs can enhance their adsorption of metals [33,34]. The adsorption of Cu and Zn on original and UV-aged polyethylene terephthalate (PET) fragments in an aqueous solution was studied [35]. It was found that the adsorption capacity of aged MPs for HMs was higher than that of the original MPs, and the adsorption capacity of aged MPs for Cu and Zn increased with radiation time [35,36]. The pH of the external environment has a certain influence on the adsorption capacity of MPs. The effect of pH on the adsorption capacity of MPs is related to the pH at the zero-point charge (pHzpc) of MPs [37]. In general, natural water pH is >pHzpc, which makes the surface of MPs negatively charged; as a result, they can electrostatically interact with metal cations [37]. When Yu et al. studied the adsorption mechanism of Cd^2+^ on PS-MPs containing hexabromocyclododecane, it was found that the pHzpc of PS-MPs was 3.8, and, when the solution pH was between 3.8 and 7.0, the adsorption capacity of PS-HBC for Cd^2+^ increased with the increasing solution pH [37]. The adsorption of heavy metal ions by MPs is affected by the type and concentration of heavy metal ions. The functional groups and chemical bonds on the surface of different MPs determine their specificity for the adsorption of heavy metal ions. Taking polyvinyl chloride and PP as examples, the amount of Zn adsorbed on the two kinds of MPs is lower than the Cu adsorbed [38]. In addition, correlations between the concentration of heavy metal ions and the adsorption capacity of MPs have also been found [17]. Heavy metal ions with concentrations similar to those of MPs are more easily absorbed and accumulated, so there are significant differences in the cumulative saturation values of different heavy metal ions on MPs.

## 3. Toxic Physiological Effects of MPs with Adsorbed HMs on Fish

To date, studies on the effects of MPs on fish have been mainly carried out under laboratory conditions [39]. Zebrafish, as a typical animal model, has been widely used to evaluate the toxic effects of MPs [40]. A large number of studies have confirmed that exposure to MPs of different particle sizes leads to different toxic physiological effects in zebrafish at different developmental stages (Table 1). High concentrations of HMs can be absorbed in MPs in the marine environment and transferred to aquatic organisms through the food chain, which may cause toxicity to fish and damage their organs (Table 2). We reviewed the toxic effects of combined MP and HM exposure on fish from three perspectives: individual, cellular, and gene expression.

### 3.1. Individual Level

**Figure 3:**
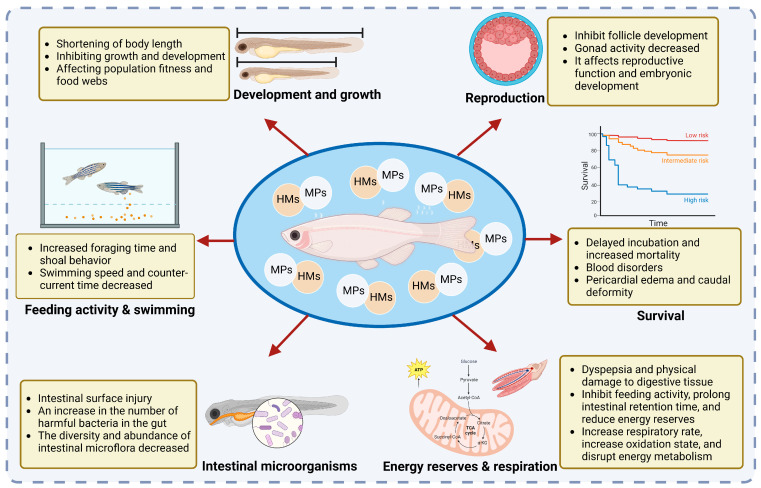
Toxic effects of heavy metals adsorbed by MPs on fish at an individual level. MPs (microplastics), HMs (heavy metals).

Fish in aquatic environments are easily affected by MPs with absorbed HMs. After ingestion, the HMs adsorbed by MPs accumulate in the gastrointestinal tract of fish and can be transferred to other organs, producing toxic effects in fish at the individual level (Figure 3).

#### 3.1.1. Survival

HMs attach to the surface of MPs, enter organisms along with the MPs through the digestive tract, and gradually accumulate in the body, ultimately having a substantial effect on the survival of fish. HMs attached to MPs have been reported to cause toxic physiological effects and even death in some organisms [59]. Statistical analysis of the cumulative mortality of blackspot seabream exposed to MPs and Cu alone or in combination revealed that heavy metal exposure during early development can severely affect fish survival, which may have implications for population health [60]. In addition, it was also found that MPs and Cu alone or in combination significantly reduced the survival rate of zebrafish larvae [61]. With increasing exposure time, the median lethal concentration decreased, and Cu toxicity increased [61]. *P. microps* juveniles caused 22% mortality at exposure containing only 0.14 mg/L MPs. The mortality rate observed with exposure containing Cd alone ranges from 33 to 100%, while the mortality range recorded with MPs and Cd mixtures ranges from 44 to 100% [62]. Therefore, the combination of microplastics and heavy metals leads to greater toxicity in fish, thus affecting the survival rate of fish. Zhang et al. showed that mortality increased with increasing MP concentration within 96 h in the combined exposure group when zebrafish were exposed to MPs and Cd [63]. However, low concentrations of MPs reduced the lethal and sublethal effects of Cd on zebrafish embryos [63]. Therefore, the adsorption of HMs by MPs may have a certain impact on the viability and health of fish populations.

#### 3.1.2. Feeding Activity and Swimming

The feeding behavior of fish can be roughly divided into three steps: foraging, predation, and intake to maintain survival, development, growth, and reproduction [64]. At present, behavioral responses have been widely used in aquatic toxicity assessments to diagnose the effects of various pollutants on the complex processes active in organisms [65]. After fish were exposed to PS for 14 days, the sensitivity of fish to prey decreased, their foraging time increased to almost twice that of the control group, and the fish remained at close distances from each other, leading to shoal behavior [55]. All of these results showed that the feeding activity of fish decreased significantly with exposure to MPs, and the hunting ability of fish was inhibited. Since swimming ability is usually affected before an organism dies, it can be used as a first indicator of the health of fish [66,67]. Santos et al. found that a high Cu concentration and microplastic association with copper significantly decreased the average speed, total travel distance, absolute turning angle, and swimming ability of zebrafish larvae and adversely affected the avoidance behavior of zebrafish [68]. Researchers exposed juvenile European seabass to mercury alone, microplastics alone, and a mixture of both for a period of 96 h [55]. The fish exposed to the lowest and highest concentrations of MPs alone and mercury alone showed at least one altered behavioral response, including drowsiness and unstable swimming behavior [68]. In addition, signs of rapid fatigue were observed in the fish exposed to all mixtures. The swimming speed and anti-reflux time of fish decreased, and the effect of the mixed exposure was more significant [68]. *P. microps* juveniles exposed to a mixture of MPs and Cd significantly reduced predation performance, and no significant difference was observed between juveniles exposed to Cd alone at the corresponding concentration [62]. Therefore, the existence of microplastics causes heavy metals to affect the predation performance of fish, thus affecting the behavior of fish. Predation performance of the common goby (*Pomatoschistus microps*) was significantly reduced by 67% when exposed to both Cr and MPs [58]. Therefore, MPs can not only increase the accumulation of HMs in bodies but also affect feeding-related movement, avoidance behavior, and the swimming ability of fish when HMs are present (Table 3).

#### 3.1.3. Energy Reserves and Respiration

Fish mainly obtain energy and nutrients through feeding. According to previous studies, the ingestion of MPs by fish can significantly inhibit the growth and energy reserves of fish [69]. This may be because fish resist the harsh environmental conditions by reducing growth and increasing metabolic demands to maintain normal physiological functions [70]. Zebrafish larvae exposed to fluorescent MPs accumulated fluorescent particles in their gut, and the ingestion of plastic particles by the organisms could reduce feeding, leading to a decreased energy intake [71]. Yin et al. found a slight decrease in crude protein and crude fat in fish after exposure to PS, indicating that PS altered the nutritional composition of fish [69]. PS can also cause a stress response, dyspepsia, and physical damage to the digestive tissues in fish, leading to changes in energy allocation [69]. In addition, the researchers found that a 1% absorption of MPs by fish can reduce the total energy reserve of fish by approximately 30%, mainly due to a reduction in fish lipid reserves [70]. MPs can alter not only energy allocation but also nutrient composition, affecting energy storage in fish. One study found that the presence of MPs in water increased mercury concentrations in the gills and liver of *Dicentrarchus labrax* juvenile [72]. MPs and mercury, alone or in combination, cause oxidative stress in both organs, and it is thought that MPs that attach to fish gills and skin cause oxygen depletion [72]. Therefore, MPs have a significant impact on both energy metabolism and respiratory exchange in fish.

#### 3.1.4. Intestinal Microorganisms

The intestinal microbiota forms a complex and dynamic system with highly variable but strong environmental adaptability, and its equilibrium state is related to the health status of fish. The intestinal tract is an important way through which all types of toxins enter fish [73]. Yan et al. found that MPs increased the accumulation of Cd, Pb, and Zn in the intestines of marine medaka (*Oryzias melastigma*), and the intestinal microflora of the fish changed significantly after exposure to a combined solution of MPs and HMs (Cd, Pb, and Zn) [74]. The diversity and abundance of intestinal microflora decreased significantly after MPs treatment but increased after HMs (Cd, Pb and Zn) and MPs-HMs treatment [74]. Some researchers also found that Cu and MPs exposure changed the diversity and structure of intestinal microorganisms [53]. Exposure to high concentrations of Cu and PS increased the number of harmful bacteria and decreased beneficial bacteria, resulting in an obvious proliferation of intestinal epithelial goblet cells, intestinal villus atrophy, and intestinal mucosal cell degeneration [53]. For example, exposure to Cu and PS increased the abundance of *Proteus* sp. and decreased the abundance of *Streptomyces* sp. in the intestinal tract, which may be related to metabolic disorders and intestinal diseases [75]. Other studies have shown that, in fish, toxic substances such as MPs and HMs are easily adsorbed on villi surfaces and increase the permeability of cell membranes by reacting with phospholipids on the cell membrane [76]. They cause damage to the surface of the intestinal tract and cause a series of inflammatory reactions. In short, MPs can introduce more HMs into the intestinal tract, causing changes in intestinal microbiota and having a certain impact on the body.

#### 3.1.5. Development and Growth

The growth and development of fish exposed to MPs and HMs can be inhibited to varying degrees. It has been shown that the average heart rate of zebrafish increased after combined exposure to low concentrations of MPs (0.05 and 0.1 mg/L) and Cd relative to cadmium exposure alone, while the average heart rate decreased after combined exposure to high concentrations of MPs (10 mg/L) and Cd [63]. The uptake of HMs by MPs during the early developmental stages of aquatic organisms may be prevented by embryonic villi, reducing the bioavailability of HMs in the embryo and, thus, their toxicity [63,77]. After the exposure of zebrafish larvae to Cu, MPs, and Cu + MPs, the body length of zebrafish larvae was significantly shortened at 14 dpf, and the growth of zebrafish larvae was inhibited, which may have occurred due to the accumulation of MPs in the intestines and false satiety in zebrafish larvae, resulting in a decrease in food intake [78]. Similarly, exposure of fish embryos to Cu or a mixture of MPs and Cu was found to result in hatching inhibition [61]. The exposed group of zebrafish larvae showed an increase in heart rate with increasing Cu concentration [78]. Co-exposure to high concentrations of Cu and MPs resulted in the highest heart rates in zebrafish larvae relative to controls. Yellow seahorse in PE, 0.05 mg/L of Cu, 0.01 mg/L of Cd, and 0.05 mg/L of Pb exposure leads to impaired body length, body weight, growth rate, and survival rate, but the separate exposure MP group and the control group had no significant differences between any parameters. So the effect of MPs on yellow seahorse growth is caused by the accumulation of HMs rather than by MPs themselves [79]. Therefore, both microplastics and heavy metals can affect fish growth and health, and the combined exposure of microplastics and heavy metals may enhance their toxicity.

#### 3.1.6. Reproduction

When fish are exposed to MPs and HMs, their reproductive functions are greatly affected. During gonadal development, Cd, Pb, and Zn and MPs, Cd, Pb, and Zn treatment can lead to follicular follicles and follicular atresia and can change the expression levels of genes related to the hypothalamus–pituitary–gonad (HPG) axis [74]. The relative proportion of primary oocytes decreased in Cd, Pb, and Zn and MPs, Cd, Pb, and Zn groups, and Cd, Pb, and Zn and MPs, Cd, Pb, and Zn treatment changed the gene expression of the HPG axis in female and male marine medaka (*Oryzias melastigma*) [74]. It was found that reproductive disorders were mainly caused by HMs, and combined treatment with MPs and Cd, Pb, and Zn did not increase the risk of gonadal developmental abnormalities in marine medaka (*Oryzias melastigma*) [74]. Rochman et al. found that a combination of MPs and persistent organic pollutants affected the endocrine system of Japanese medaka (*Oryzias latipes*), resulting in the abnormal proliferation of germ cells [80]. Therefore, HMs adsorption by MPs have an impact on the reproduction of fish.

### 3.2. Cellular Level

**Figure 4:**
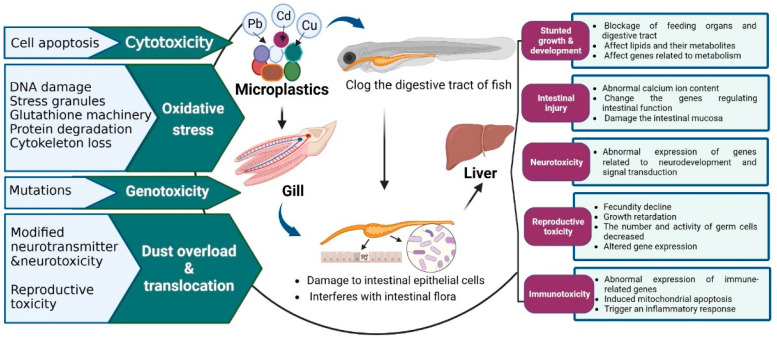
Transfer and toxic effects of microplastics in fish. MPs (microplastics).

The damage caused by MPs and HMs to fish at both the individual and cellular levels is closely related to their related gene expression effects. MPs and HMs affect the expression of different genes in fish to varying degrees (Figure 4).

#### 3.2.1. Cytotoxicity

Current studies have shown that MPs can cause a certain degree of cell damage even at low concentrations [81]. Guimaraes et al. conducted a 20-day exposure experiment on grass carp and found that even the lowest concentration (0.04 ng/L) was sufficient to cause DNA breakage and damage to juvenile grass carp throughout the exposure period and to induce mutagenic and cytotoxic effects on red blood cells [82]. HMs easily penetrate biofilms and accumulate in fish, leading to oxidative stress and other cytotoxic effects, resulting in cell damage [83]. In addition to affecting the normal physiological functions of aquatic organisms, in the presence of HMs, MPs can also cause a series of toxic effects related to HMs. At the cellular level, MPs can damage the cellular structure and lead to apoptosis [84]. The joint exposure of MPs and Cu led to the strong cytoplasmic staining and nuclear aggregation of some goldfish liver cells, and the cytoplasm was concentrated, and the cell boundary was blurred. This suggests that combined exposure of MPs and Cu leads to a more severe hepatopancreas injury in goldfish [75]. Lu et al. exposed zebrafish to MPs containing Cd and PS for 3 weeks and found that the presence of MPs could increase the accumulation of Cd in the gills, intestines, and liver of zebrafish [85]. Moreover, under Cd exposure and high concentrations of MPs, most zebrafish gills, intestines, and liver cells showed varying degrees of damage [85]. By identifying more than 200 cell populations in zebrafish, the researchers found that PS exposure had the greatest effect on macrophages, while Pb exposure mainly affected intestinal cells [76]. Therefore, the combined exposure of microplastics and heavy metals can cause cytotoxicity in fish, thus causing damage to the bodies of fish.

#### 3.2.2. Oxidative Damage

A large number of studies have shown that MPs and HMs can cause significant oxidative damage to fish. The antioxidant defense system plays an important role in organisms; it can maintain appropriate levels of free radicals and prevent oxidative damage [86]. Ingested MPs mainly accumulated in the gut of fish. In fish treated with MPs, the antioxidant enzymes catalase (CAT) and superoxide dismutase (SOD) in the gut were significantly increased, and the content of glutathione in the intestinal tissue was increased, leading to an inflammatory response and oxidative damage in the intestinal tissue [87,88]. At the same time, HMs such as Cd and Hg can inhibit the activities of the antioxidant enzymes CAT, SOD, and glutathione peroxidase, causing oxidative damage [89]. In addition to MP toxicity in fish, the combined toxic effects of MPs and HMs have received much attention. It was found that the co-exposure of MPs and Cu led to significantly up-regulated CAT expression and significantly down-regulated SOD expression in the goldfish hepatopancreas, indicating that the combined exposure of MPs and Cu induced more severe oxidative stress and destroyed the balance of the antioxidant defense system in goldfish [75]. Lu et al. exposed zebrafish to MPs and Cd alone or in combination. Their results showed that the effect of the combined treatment of Cd and MPs on glutathione was greater than that of Cd alone, and the effect was enhanced with increasing MP concentration [85]. Additionally, combined exposure to Cd and MPs caused a significant decrease in SOD activity in the gut and gills of fish with increasing MP concentration [85]. In addition to the oxidative damage caused by MPs and Cd in fish, these pollutants can also cause harm via other pathways. One possibility is that when MPs transport Cd into an organism, the tissue accumulation of MPs and the adsorption of Cd result in a microenvironment that continuously releases the metal [85]. Therefore, MPs combined with HMs can cause oxidative damage to fish through a variety of ways, affecting body health.

#### 3.2.3. Inflammatory Response

The liver is the main storage site for MPs and HMs in fish and the main site for cytotoxic effects such as inflammatory responses in fish [72,90]. Yu et al. found that B cells and T cells of the adaptive immune response in zebrafish were also affected by PS and Pb exposure, while B cells and T cells were affected by MAPK signaling pathways regulating inflammation and innate immunity [76]. Cheng et al. used PS nanoplastic particles and micron-sized PS particles to study the effect of MPs on liver inflammation in zebrafish larvae [45]. They found that the smaller the MP particles were, the higher the degree of aggregation of neutrophils in the abdomen of larvae, the higher the apoptosis rate of macrophages, and the greater the degree of liver inflammation [45]. HMs can increase the accumulation of ROS in immune cells at damaged sites, forming a persistent inflammatory and oxidative environment. When Qin et al. exposed zebrafish to Cd and micron-sized PS particles, with Cd treatment, the number of neutrophils decreased by 25.56%, and the Cd content in juvenile fish decreased [91]. However, when the fish were co-exposed to Cd and 100 nm PS particles, the content of Cd in juvenile fish decreased, and the expression of neutrophils remained high [91]. Liu et al. conducted Cu exposure experiments on swamp eel (*Monopterus albus*) and found that Cu could regulate immune response, resulting in the release of more proinflammatory cytokines and producing inflammatory responses [92]. In addition, a study on young *Channa argus* found that the presence of Cd did not affect the expression of the *IL-1β* gene, but, when 0.5 μm of MPs was present in water, they had an antagonistic effect, reducing the effect of the MPs on the immunity of the organisms [93]. However, the presence of 0.5 μm of MPs in water will promote the effect of 0.5 μm of MPs on the body’s immunity [90]. Therefore, MPs and Cd can affect the expression of immune-related genes to different degrees.

#### 3.2.4. Neurotoxicity

Exposure to MPs can induce neurotoxicity through the disruption of lipid peroxidation and interferences with nerve-related enzymes in fish. Bhagat et al. showed that MP accumulation in fish can lead to the inhibition of various neurotransmitters [94]. Because acetylcholinesterase (AchE) provides information about potential neuromuscular cholinergic damage, it is used as a primary indicator of neurotoxicity [95]. It has been reported that both MPs and Cd can produce neurotoxicity in fish by reducing the activity of AChE [62]. Inhibition of AChE activity significantly increases the level of acetylcholine in synaptic spaces, hinders nerve information transmission, and destroys the functions of the nervous system, leading to serious motor dysfunction and behavioral abnormalities [62,96]. MPs can also cause additional damage to fish through their interactions with HMs. Predator avoidance and social behavior depend on a healthy and functional nervous system. When fish are exposed to Cu, these systems are destroyed and behavioral disorders occur, reducing the adaptability and survival of fish stocks [97]. In addition, there was a positive correlation between AChE and hatching [61]. A decrease in embryonic motility caused by the inhibition of AChE activity may be the reason for the low hatching rate [62,98]. In addition, MPs can inhibit the neurodevelopmental toxicity of Hg during embryonic development of zebrafish depending on its size [99]. Santos et al. also found that MPs and copper alone or in mixtures regulate the synthesis and transport of dopamine in the exposed zebrafish brain [100]. Dopamine may function as a neurotoxin. Dopamine at high levels produces ROS, free radicals, and hydroquinone during oxidation, which may play a role in the MP-induced apoptosis of fish brain cells, resulting in neurotoxicity [100]. The common goby (*Pomatoschistus microps*) significantly inhibited AChE activity by up to 31% when exposed to Cr and MPs at the same time, higher than the inhibition induced by MPs alone [58]. The effect of combined exposure to heavy metals and microplastics on AChE activity is, therefore, a matter of serious concern, as they may lead to reduced individual performance and eventual death, with negative effects on population health.

#### 3.2.5. Metabolism

Exposure to MPs, which is thought to interfere with lipid and energy metabolism in the fish liver, also altered the metabolomic profile of fish. Lu et al. found that exposure to PS caused significant elevations in triglycerides and fatty acids and induced changes in choline, phosphocholine, and cholesterol, which disrupted lipid metabolism in fish [101]. In addition, it led to a reduction in branched-chain amino acids, such as isoleucine, leucine, valine, and lysine, further altering energy metabolism [102]. On the other hand, a high intake of MPs can affect fish feeding behavior as well as normal food absorption, and changes in food absorption can also lead to changes in lipid and energy metabolism [69]. Furthermore, the diversity of the gut microbiota in male marine medaka (*Oryzias melastigma*) was significantly increased after combined MPs, Cd, Pb, and Zn exposure. Predictions of gut microbiota functions showed that lipid metabolism, xenobiotic degradation, and metabolism, as well as the metabolism of terpenoids and polyketide drugs, were significantly upregulated, while carbohydrate metabolism, nucleotide metabolism, and amino acid metabolism pathways were significantly downregulated [74]. However, combined MPs, Cd, Pb, and Zn exposure significantly reduced membrane transport, while it significantly increased the metabolism of terpenoids and polyketide compounds compared to Cd, Pb, and Zn exposure [74]. In addition, PS promoted catabolic processes, which are the pathways of amino acid metabolism, purine metabolism, and steroid hormone biosynthesis in Cd-treated zebrafish larvae [91]. This suggests that the combined metabolic effects of MPs and HMs are size-dependent.

### 3.3. Molecular Level

#### Gene Expression

In previous studies, MPs and Cd affected the expression of antioxidant enzymes in organisms, which led to a decline in antioxidant capacity and an increase in oxidative stress, and the antioxidant capacity of fish was affected, which may have inhibited the expression of immune-related genes [34,96]. The co-exposure of MPs and Cu significantly up-regulated the relative expressions of pro-apoptotic genes such as *Caspase 8*, *Caspase 9*, and *Bax*, while significantly down-regulating the anti-apoptotic gene *Bcl-2* in the goldfish hepatopancreas [75]. Therefore, combined exposure of MPs and Cu may induce excessive apoptosis and may lead to normal tissue cell death and inhibit tissue repair. Santos et al. exposed zebrafish embryos to MPs and copper for 14 days and found that genes related to DNA methyltransferase, neuronal proliferation, neurogenesis, and motor neuron development were downregulated, resulting in neurological defects in the early life stages of zebrafish [98]. It has been demonstrated that changes in overall behavioral characteristics are further associated with the disordered mRNA expression patterns of related key genes [103]. In addition, the expression of *npc2*, *apoa1a*, *apoa*, and *apoa4a*, which are involved in cholesterol efflux and steroid metabolism, also changed after combined exposure to MPs and Pb [76]. More interestingly, MP regulation of the expression of these genes is not isolated but has a cascade effect [104,105]. Liu et al. found that the upregulation of antioxidant enzyme-related genes caused by microplastic exposure also affected the expression of some genes in the central nervous system and those associated with retinal development [102]. Therefore, further studies on the effect of MPs on fish gene expression are needed to facilitate the control of microplastic pollution in the future, which is an urgent problem to be solved.

## 4. Conclusions and Prospects

**Figure 5:**
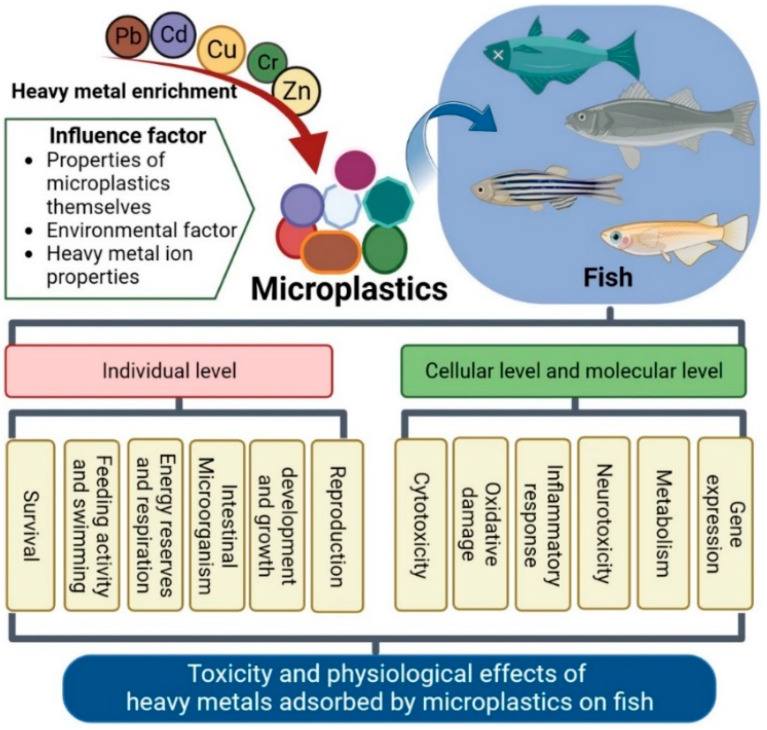
Factors influencing the adsorption of heavy metals by microplastics and toxic effects at individual, cellular, and molecular levels in fish.

At present, MPs are a class of environmental pollutants of emerging concern. These small plastic particles are widely distributed in almost all types of aquatic habitats around the world. All types of fish are negatively affected by microplastic exposure. In addition, the HM adsorption by MPs has a complex, toxic effect on fish (Figure 5). Therefore, an in-depth study on the toxicity mechanisms of MPs containing adsorbed HMs on fish could provide valuable information for environmental protection and pollution prevention.

To reduce microplastic pollution, it is desirable to control the sources, reduce the use of disposable plastic products, develop and promote recyclable and biodegradable alternatives, and formulate laws and regulations to control plastic use [94]. In future research, scientists should further clarify the ecotoxicological hazards of MPs, continue to develop efficient and reliable MP detection techniques, and unify classification and testing standards. In addition, the treatment efficacy of microplastic point sources, such as sewage treatment plants, should be improved, and new high-efficiency remediation processes for removing MPs should be developed to prevent further harm from the conversion of large MPs into nanoplastics in aquatic environments.

## Figures and Tables

**Table 1:** Toxic effects of different particle size plastics on zebrafish at different developmental stages.

Types of Microplastics	Particle Size	Exposure Concentration	Exposure Time	Toxic Effect	References
Embryo (0–5 d)					
PET	150 mm	20 mg/L	3 d	Heart rate increases, blood flow rate increases, hatching rate decreases	[41]
PS	10 μm	200 particles/mL	5 d	Oxidative stress, developmental deformities, and decreased survival rate	[42]
PS	42 nm	10 mg/mL	5 d	Oxidative stress, inflammation, and apoptosis increased	[43]
Juvenile (5–90 d)					
PS	25 nm	20 mg/L	2 d	Glucose metabolism is disturbed, and behavior is inhibited	[44]
PS	50 nm, 100 nm	0.1 mg/L, 0.5 mg/L, 2 mg/L, 10 mg/L	3 d	Oxidative stress, liver inflammation, liver immunotoxicity	[45]
PE	50 nm, 45 μm	1 mg/L	120 h	Oxidative stress, inhibited behavior, and shortened body length	[46]
Adult fish					
PE	50 μm, 100 nm	100 μg/L	14 d	Oxidative damage to the central nervous system, intestinal flora imbalance, stimulate immune response	[47]
PE	30 μm	1000 μg/L	7 d	Immunotoxicity, intestinal inflammation	[48]
PE	80 nm, 8 μm	10 μg/L, 1 mg/L	21 d	Intestinal flora disorder, intestinal inflammation	[49]
PE	3–12 µm	260 mg/L	21 d	Oxidative stress, metabolic changes, DNA damage	[50]
PE	10–22 μm, 45–53 μm, 90–106 μm, 212–250 μm, 500–600 μm	2 mg/L	4 d	Intestinal injury, neurotoxicity	[51]
PS	90% < 90 μm, 10% < 25 μm	100 μg/L 1000 μg/L	20 d	Immunotoxicity, lipid metabolism changes	[52]

PE (polyethylene), PS (polystyrene),PET (polyethylene terephthalate).

**Table 2:** Toxic effect of microplastics with adsorbed heavy metals on fish.

Species of Fish	Types of MPs	Particle Size	MPs Concentration	Types of HMs	HMs Concentration	Exposure Duration	The Effects of Biological Toxicity	References
Nile tilapia (*Oreochromis niloticus*)	PS	0.1 μm	1 mg/L	Cu	0.5 mg/L, 1 mg/L, 2 mg/L	24 h	1. Caused serious pathological changes in the internal organs. 2. Increased the number of harmful bacteria in the gut of Nile tilapia and decreased the immunity.	[53]
Catfish (*Clarias gariepinus*)	Polyamide 12, PLA	N.A.	N.A.	Cu	0.050 mg/L	3 months	1. The concentration of Cu and Pb in gills was the highest, followed by that in the liver and intestine. 2. The microflora become disordered, reducing the immunity and causing potential vibrio infection.	[54]
				Pb	0.060 mg/L			
European seabass (*Dicentrarchus labrax*)	Polymer microspheres	1–5 μm	5–28%	Hg	45–53%	96 h	1. Affects the behavioral responses. 2. Increases the bioconcentration of Hg in the gills and bioaccumulation in the liver.	[55]
Zebrafish (*Danio rerio*)	PS	1–5 μm	2 mg/L	Cu	25 μg/L	30 d	Resulting in increased membrane lipid oxidation, SOD activity decreased, DNA damage and so on.	[56]
Discus fish (*Symphysodon aequifasciatus*)	PS	32–40 μm	50 μg/L, 500 μg/L	Cd	50 μg/L	30 d	Severe oxidative stress was observed.	[57]
Goby (*Pomatoschistus microps*)	PE	1–5 μm	0.184 mg/L	Cr	5.6 mg/L, 8.4 mg/L, 12.6 mg/L, 18.9 mg/L, 28.4 mg/L	48 h	Inhibited 31% of the acetylcholinesterase activity.	[58]

MPs (microplastics), HMs (heavy metals), PS (polystyrene), PE (polyethylene), PLA-PA (polyamides), Cu (copper), Pb (lead), Hg (mercury), Cd (cadmium), Cr (chromium).

**Table 3:** Effect of heavy metal adsorption by microplastics on fish behavior.

Species of Fish	Types of MPs	Particle Size	MPs Concentration	Types of HMs	HMs Concentration	Exposure Time	Effects on Fish Behavior	References
Zebrafish (*Danio rerio*)	polymer microspheres	1–5 µm	2 mg/L	Cu	60 μg/L, 125 μg/L	14 d	Decreased the average speed, travel distance, and absolute turning angle. Adversely affected their swimming ability. Affected the avoidance behavior. Larvae did not respond to adverse stimuli.	[68]
European seabass *(Dicentrarchus labrax)*	polymer microspheres	1–5 µm	5–28%	Hg	45–53%	96 h	Reduced swimming speed and survival time.	[55]
Grass carp *(Ctenopharyngodon idellus)*	PS	5 μm	700 μg/L	Cd	100 μg/L	24 h, 48 h	The swimming speed increased first and then decreased. The behavioral mechanisms were altered. Reduce their exposure to pollutants by reducing their activity levels and metabolic rates.	[67]

MPs (microplastics), HMs (heavy metals), PS (polystyrene), Cu (copper), Hg (mercury), Cd (cadmium).

## Data Availability

Not applicable.

## Abbreviations

MPs, microplastics; HMs, heavy metals; PS, polystyrene; PAE, phthalate esters; POM, polyoxymethylene; PVC, polyvinyl chloride; PP, polypropylene; PLA-PA, polyamides; PE, polyethylene; PET, polyethylene terephthalate; pHzpc, PH at zero-point charge; HPG, hypothalamus–pituitary–gonad; CAT, catalase; SOD, superoxide dismutase; AchE, acetylcholinesterase.
